# A comparative study of characteristics in diploid and tetraploid *Anoectochilus roxburghii*

**DOI:** 10.3389/fnut.2022.1034751

**Published:** 2022-11-07

**Authors:** Xiaoling Huang, Kunxi Ouyang, Yongzhi Luo, Guohong Xie, Yuesheng Yang, Junjie Zhang

**Affiliations:** ^1^Guangdong Key Laboratory for Innovative Development and Utilization of Forest Plant Germplasm, Guangzhou, China; ^2^Guangdong Province Research Center of Woody Forage Engineering Technology, Guangzhou, China; ^3^College of Forestry and Landscape Architecture, South China Agricultural University, Guangzhou, China; ^4^Shatoujiao Forest Farm, Shenzhen, China; ^5^Southern Medicine Research Institute of Yunfu, Yunfu, China

**Keywords:** *Anoectochilus roxburghii*, tetraploid, kinsenoside, phenotype, biochemical

## Abstract

Artificial induction of polyploidy is an efficient technique for improving biological properties and developing new varieties of many plants. In this study, we analyzed and compared differences in characteristics (morphological and biological) of diploid and tetraploid *Anoectochilus roxburghii* plants. We found significant differences between tetraploid plants and their diploid counterparts. The tetraploid plants exhibited dwarfing and stockiness. They were also bigger and had more voluminous roots and larger stomata than the diploid plants. Moreover, the biochemical analyses showed that the contents of some amino acids and minerals elements were significantly higher in tetraploid plants. The chlorophyll content of the leaves exhibited no definitive changes, but the photosynthetic performance was higher in the tetraploid plants. In addition, contents of major bioactive compounds, such as kinsenoside and some flavonoids, were enhanced in tetraploids. This is the first detailed analysis of characteristics in diploid and tetraploid *A. roxburghii* plants. The results may facilitate breeding programs with the species.

## Introduction

*Anoectochilus roxburghii* (Wall.) Lindl, commonly known as Jinxianlian, belongs to the Orchidaceae family and is naturally distributed in many parts of a broad zone of the tropics, encompassing India, the Himalayas, southern China, large tracts of Southeast Asia and Hawaii (1). As a valuable element of Chinese herbal medicine, this species is frequently used in medical and health products in China and Asian countries (2–4). It is also often used as an indoor plant as it is highly ornamental (5). In recent years industrial-scale use of *A. roxburghii* has grown as a result of its wide application in many fields, such as medicine, healthcare and so on (6). Most raw material of the plant for industrial processing is obtained by artificial cultivation, as wild resources cannot meet the market demand (7–9).

Polyploid plants, either naturally occurring or generated with chromosome doubling techniques, are used in many breeding programs for medicinal plants to increase biomass and enhance desirable traits (10–12). Chromosome doubling is often accompanied by substantial changes in morphology and metabolic profiles (13, 14). It also has generally positive effects on stress resistance (15). Thus, polyploidy induction can be used to develop superior varieties. In this study, the morphology and biochemical characteristics of diploid and induced tetraploid *A. roxburghii* were compared. The ploidy level effects on the plant’s ability to resist high temperature stress were also assessed.

## Results

### Detection of ploidy level and morphological traits of diploid and tetraploid plants

**FIGURE 1:**
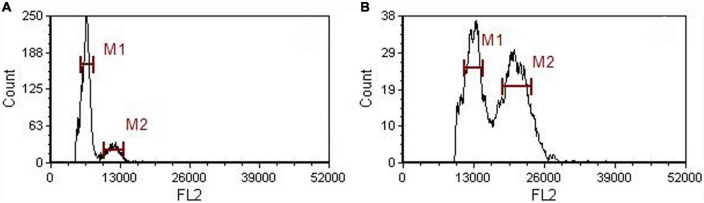
Histograms showing the results of flow cytometric analysis of diploid **(A)** and tetraploid **(B)** *A. roxburghii* plants.

**FIGURE 2:**
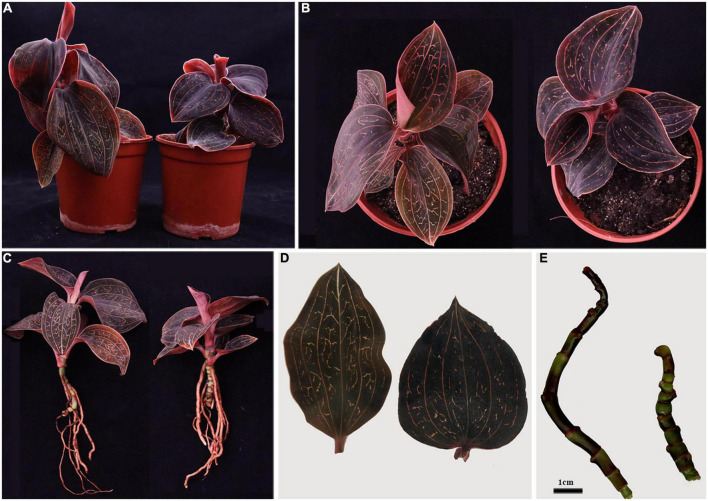
Morphological characteristics of diploid and tetraploid *A. roxburghii*: whole plants **(A–C)**, leaves **(D)** and stems **(E)**. Diploids on the left and tetraploids on the right in each picture.

Flow cytometric peaks of the diploid mother and tetraploid offspring plants (at channels 13,000 and 6,500, respectively) confirmed their ploidy levels (Figure 1). We assessed various morphological characteristics of the diploid and tetraploid plants when they were 1 year old, and observed significant differences in some of them (Figure 2). Tetraploid plants had higher biomass (both fresh and dry weight), although the diploids were significantly taller, due to longer internodes. The tetraploid plants had thicker stems than the diploids, and shorter but broader and hypertrophic leaves. The tetraploid plants also produced more roots and had significantly higher total root length, root surface area, and root volume (Table 1).

**TABLE 1:** Morphological characteristics of diploid and tetraploid *A. roxburghii.*

Characters	Diploid	Tetraploid
Fresh weight (g)	13.0 ± 2.63^a^*	16.31 ± 2.99^a^
Dry weight (g)	1.20 ± 0.31^a^	1.32 ± 0.20^a^
Plant height (cm)	12.71 ± 3.05^a^	7.33 ± 1.22^b^
Stem diameter (mm)	4.80 ± 0.49^a^	5.24 ± 0.30^a^
Internode length (cm)	1.12 ± 0.22^a^	0.68 ± 0.17^b^
Leaf length (cm)	7.17 ± 0.75^a^	5.83 ± 0.28^b^
Leaf width (cm)	4.98 ± 0.46^a^	5.38 ± 0.36^a^
Leaf area (cm^2^)	26.35 ± 4.14^a^	22.57 ± 1.65^a^
Leaf index (length/width)	1.44 ± 0.05^a^	1.09 ± 0.12^b^
Leaf thickness (mm)	0.62 ± 0.02^b^	1.10 ± 0.12^a^
Root length (cm)	37.38 ± 14.35^b^	78.12 ± 10.02^a^
Root diameter (mm)	1.50 ± 0.20^a^	1.93 ± 0.25^a^
Root surface area (cm^2^)	20.23 ± 9.96^b^	56.24 ± 8.14^a^
Root volume (cm^3^)	0.78 ± 0.44^b^	2.76 ± 0.71^a^

*Means followed by the same letter in the same row are not significantly different from each other.

### Comparison of free amino acid and mineral element contents of diploid and tetraploid plants

*Anoectochilus roxburghii* has traditionally been considered to be one of the most valuable medicinal plants because it is rich in amino acids, minerals and so on (8). The *amino acids* are necessary for metabolic processes, the transport and storage of all nutrients (16). Minerals play important roles in human health, not only affecting the enzyme activities, but also influencing the accumulation of metabolites (17). The types of free amino acids identified in diploids and tetraploids plants were identical, but the detected amounts differed (Table 2). Two of the most abundant amino acids in *A. roxburghii* were aspartate (Asp) and glutamate (Glu) and the average content was 2,254 μg/g and 1,067 μg/g in diploids plants, respectively. Contents of these two amino acids were greater in tetraploid than diploid plants. In addition to these two amino acids, there were some other amino acids that were more abundant in tetraploids plants, such as serine (Ser) and glycine (Gly). While there were some amino acids were more abundant in diploids, such as phenylalanine (Phe) and histidine (His). Tetraploid plants had higher concentrations of Ca, Mg, and Zn than diploids. The largest difference in concentration recorded between the plants was for Ca, which was about twice as high in tetraploids than diploids. Concentrations of Mn and Cr were lower in tetraploid plants (Table 3), but no significant differences in contents of the other measured elements were detected.

**TABLE 2:** Free amino acid content of diploid and tetraploid *Anoectochilus roxburghii.*

Amino acid (μg/g)	Diploid	Tetraploid
P-Ser	195.92 ± 27.81^a^*	197.88 ± 6.86^a^
PEA	13.08 ± 2.82^b^	42.17 ± 6.49^a^
Asp	2254.47 ± 48.82^b^	13562.55 ± 359.80^a^
Thr	38.13 ± 9.45^a^	38.51 ± 5.10^a^
Ser	110.71 ± 11.68^b^	150.82 ± 15.97^a^
AspNH2	12.07 ± 1.44^b^	684.07 ± 27.13^a^
Glu	1066.92 ± 26.94^a^	1474.79 ± 68.53^b^
GluNH2	245.94 ± 15.44^b^	401.01 ± 17.94^a^
Gly	4.38 ± 1.59^b^	27.0 ± 5.51^a^
Ala	260.24 ± 9.94^a^	285.58 ± 19.96^a^
Val	31.96 ± 6.93^a^	28.03 ± 3.41^a^
Met	3.26 ± 0.81^a^	1.52 ± 0.27^b^
Ile	19.73 ± 3.68^a^	16.97 ± 3.44^a^
Leu	9.84 ± 1.09^a^	11.37 ± 0.35^a^
Tyr	11.52 ± 1.67^a^	9.97 ± 1.44^a^
Phe	35.59 ± 2.13^a^	24.05 ± 4.59^b^
b-Ala	5.05 ± 0.44^b^	21.51 ± 5.02^a^
g-ABA	32.29 ± 3.04^b^	89.33 ± 2.39^a^
Hylys	2.98 ± 0.84^a^	1.32 ± 0.53^b^
Orn	3.11 ± 0.44^b^	16.97 ± 3.08^a^
Lys	31.88 ± 5.57^a^	31.40 ± 5.11^a^
His	67.46 ± 8.07^a^	37.32 ± 5.79^b^
Arg	16.32 ± 2.59^b^	78.69 ± 7.33^a^

*Means followed by the same letter in the same row are not significantly different from each other. P-Ser, PhosphoSerine; PEA, Phenylethylamine; Asp, Aspartate; Thr, Threonine; Ser, Serine; AspNH2, Isoasparagine; Glu, Glutamate; GluNH2, Isoglutamine; Gly, Glycine; Ala, Alanine; Val, Valine; Met, Methionine; Ile, Isoleucine; Leu, Leucine; Tyr, Tyrosine; Phe, Phenylalanine; β-Ala, β-Alanine; γ-ABA, γ-Aminobutyric Acid; Hylys, 5-Hydroxylysine; Orn, Ornithine; Lys, Lysine; His, Histidine; Arg, Arginine.

**TABLE 3:** Mineral elements detected in diploid and tetraploid *Anoectochilus roxburghii.*

Mineral elements	Diploid	Tetraploid
K (mg/Kg)	30140.32 ± 1037.19^a^*	25911.55 ± 3955.27^a^
Ca (mg/Kg)	250.33 ± 28.16^b^	578.65 ± 14.94^a^
Mg (mg/Kg)	140.10 ± 0.90^b^	167.93 ± 4.08^a^
Fe (mg/Kg)	230.51 ± 94.83^a^	233.45 ± 53.35^a^
Zn (mg/Kg)	4.78 ± 0.12^b^	6.67 ± 0.15^a^
Mn (mg/Kg)	7.70 ± 0.09^a^	6.0 ± 0.13^b^
Cu (mg/Kg)	3.21 ± 0.24^a^	3.39 ± 0.98^a^
Co (μg/Kg)	149.34 ± 44.31^a^	142.75 ± 19.25^a^
Cr (μg/Kg)	4052.50 ± 58.60^a^	3491.10 ± 53.53^b^
Ni (μg/Kg)	1256.6221 ± 566.88^a^	866.06 ± 124.10^a^

*Means followed by the same letter in the same row are not significantly different from each other.

### Determination of main bioactive secondary metabolite production

Kinsenoside and flavonoids, the main bioactive compounds in *A. roxburghii*, are often used as quality control markers (8). Narcissin, isorhamnetin, rutin, and quercetin are the characteristic flavonoids (8, 18). Thus, we examined contents of these five compounds in leaves, stems and roots of diploid and tetraploid plants (Table 4). They were mainly found in leaves. The kinsenoside concentration was non-significantly higher in tetraploid than diploid leaves, and the rutin level was slightly higher in tetraploid plants. Contents of the other three flavonoids were slightly higher in diploid plants. The results suggest that chromosome doubling had little impact on the main bioactive secondary metabolites.

**TABLE 4:** Content of bioactive compounds in diploid and tetraploid *Anoectochilus roxburghii.*

Ploidy	Leaves					Stems					Roots				
			
	Kinse- noside (mg/g)	Narcissin (μg/g)	Isorha- mnetin (μg/g)	Quercetin (μg/g)	Rutin (μg/g)	Kinse- noside (mg/g)	Narcissin (μg/g)	Isorha- mnetin (μg/g)	Quercetin (μg/g)	Rutin (μg/g)	Kinse- noside (mg/g)	Narcissin (μg/g)	Isorha- mnetin (μg/g)	Quercetin (μg/g)	Rutin (μg/g)
Diploid	104.17 ± 6.56^a^*	352.18 ± 167.23^a^	13.14 ± 3.47^a^	3.66 ± 1.77^a^	5.37 ± 1.63^a^	59.44 ± 7.58^a^	0.97 ± 0.36^a^	0.49 ± 0.09^a^	2.0 ± 0.30^a^	1.92 ± 0.08^b^	34.71 ± 8.56^a^	0.54 ± 0.08^a^	0.74 ± 0.30^a^	0.46 ± 0.18^a^	0.82 ± 0.38^a^
Tetraploid	115.15 ± 17.14^a^	244.10 ± 171.31^a^	4.43 ± 1.24^b^	3.31 ± 0.46^a^	7.68 ± 3.77^a^	33.45 ± 5.79^b^	1.10 ± 0.40^a^	0.32 ± 0.05^b^	0.97 ± 0.16^b^	3.77 ± 0.49^a^	22.03 ± 9.84^a^	0.39 ± 0.26^a^	0.35 ± 0.11^a^	0.76 ± 0.30^a^	0.90 ± 0.56^a^

*Means followed by the same letter in the same column are not significantly different from each other.

### Comparison of chlorophyll contents, stomatal characteristics, and chlorophyll fluorescence

Chlorophyll (Chl) is the most important pigment for capturing the light required for photosynthesis in higher plants and thus plays a key role in the conversion of light energy into chemical energy needed for plant growth. Chl content was not much affected by polyploidization. Chl a and Chl b levels were higher in diploid *A. roxburghii* plants than in tetraploids, but the differences were not significant (Table 5). However, tetraploids had significantly longer and wider stomata than diploids (Table 6).

**TABLE 5:** Content of photosynthetic pigments in diploid and tetraploid *Anoectochilus roxburghii.*

Characters	Diploid	Tetraploid
Chlorophyll a (mg/g)	0.39 ± 0.10^a^*	0.31 ± 0.04^a^
Chlorophyll b (mg/g)	0.24 ± 0.04^a^	0.19 ± 0.02^a^
Chlorophyll a + b (mg/g)	0.63 ± 0.15^a^	0.50 ± 0.05^a^

*Means followed by the same letter in the same row are not significantly different from each other.

**TABLE 6:** Stomatal characteristics of diploid and tetraploid *Anoectochilus roxburghii.*

Characters	Diploid	Tetraploid
Length (μm)	49.60 ± 1.41^b^*	82.0 ± 3.55^a^
Width (μm)	11.48 ± 0.97^b^	21.67 ± 1.91^a^

*Means followed by the same letter in the same row are not significantly different from each other.

Variations in structural and physiological elements may influence plants’ photosynthetic performance and we found significant differences in chl fluorescence traits between diploid and tetraploid plants (Table 7). The maximum photochemical efficiency of PSII photochemistry (*Fv/Fm*) was significantly higher in tetraploid plants. However, Y (I) and ETR (I) were higher in tetraploid than diploid plants and stronger than Y (II) and ETR (II). We also found that polyploidy resulted in a significant reduction in NPQ.

**TABLE 7:** Chlorophyll fluorescence of diploid and tetraploid *Anoectochilus roxburghii.*

Chlorophyll fluorescence	Diploid	Tetraploid
Fv/Fm	0.77 ± 0.02^b^*	0.79 ± 0.01^a^
Y (I)	0.60 ± 0.05^b^	0.68 ± 0.04^a^
ETR (I)	23.76 ± 1.99^b^	26.75 ± 1.39^a^
Y (ND)	0.30 ± 0.06^a^	0.20 ± 0.04^b^
Y (NA)	0.10 ± 0.02^a^	0.13 ± 0.04^a^
Y (II)	0.26 ± 0.03^b^	0.34 ± 0.03^a^
ETR (II)	10.49 ± 1.08^b^	13.48 ± 1.34^a^
NPQ	2.53 ± 0.46^a^	1.56 ± 0.17^b^

*Means followed by the same letter in the same row are not significantly different from each other. Fv/Fm, maximum efficiency of PSII photochemistry; Y(I), quantum yield of PSI; ETR(I), electron transport rates of PSI; Y (ND), non-photochemical quantum yields of PSI due to donor-side limitation; Y (NA), non-photochemical quantum yields of PSI due to acceptor-side limitation; Y (II), effective photochemical quantum yield of PSII; ETR (II), electron transport rates of PSII; NPQ, non-photochemical quenching.

## Discussion

Multiple studies have shown that polyploid plants may differ morphologically, ecologically, physiologically, and cytologically from parental lines (10, 12–14). The variations have also been exploited in the development of superior varieties in breeding programs. Our results are consistent with findings that polyploidy is often associated with agronomic improvements, such as higher biomass, leaf thickness, stem diameter, and root development (12, 19). The mechanisms by which chromosome doubling produces superior traits in induced polyploid plants are not known. The increases in stem and leaf thickness may be due to increases in cell size, while genetic changes in the cells may enhance activities of key genes (19). It has also been suggested that changes in polyploid plants’ morphology may be associated with improvements in abiotic tolerance, genetic adaptability and tolerance of environmental stresses (20).

Although effects of polyploidy are not generally predictable and may be taxon-specific, doubling the chromosome number of plant species may enhance their overall nutrient contents and secondary metabolism (21, 22, 12). Accordingly, we found that concentrations of Asp (six times) and γ-ABA (more than twice) were higher in tetraploid *A. roxburghii* plants than in the diploids. Similar increases have been reported in artificial tetraploid rice (23). We also found much higher Ca contents, which may result in increased cell volumes (13), in tetraploid plants. The higher Ca levels in tetraploids may also be related to stress adaptation (24). The tetraploid leaves had significantly higher kinsenoside and rutin levels than diploid leaves, indicating that these phytochemical characteristics are strongly influenced by the plant ploidy level. Similar increases have been described in other artificial tetraploid plants, such as a higher yield of cichoric acid in tetraploid *Echinacea purpurea* (12) and a 47.7% increase in rubber concentration in tetraploid *Taraxacum kok-saghyz* (25) tetraploids.

Stomatal lengths and widths have often been used as morphological markers for identifying putative polyploid plants (26). We found that tetraploid *A. roxburghii* plants had significantly longer and wider stomata than the diploids, in accordance with previous findings that diploids *Taraxacum kok-saghyz* plants have lower stomatal length and width than counterparts with higher ploidy levels (25). Larger leaves and greater numbers of stomata may enhance photosynthesis, thereby indirectly increasing the plants’ environmental adaptability and improving their abiotic tolerance (13).

Chl is a key player in interactions with light during the entire life cycle of plants. We detected no clear ploidy level-associated variation in *A. roxburghii* leaves. No such variation had been detected in *Urgenia indica* either (27). However, chromosome doubling has reportedly increased chl contents of some species, e.g., *Miscanthus*× *giganteus* (13), and decreased those of *Juncus effusus* (28). Chl fluorescence measurements are mainstays of studies of photosynthetic regulation and plants’ environmental responses because of their sensitivity, convenience, and non-intrusive nature (29). *Fv/Fm*, indicating the amount of absorbed energy trapped in PSII reaction centers, is an excellent parameter for monitoring temperature stress (30). We found that tetraploid *A. roxburghii* plants had higher *Fv/Fm* values than diploids, indicating that tetraploid plants were more resistant to heat. NPQ reflects the amount of energy from photosynthetic electron transport that is not used but dissipated harmlessly as heat from PSII antennae, which is a key damage-avoidance mechanism in plants (29). We found that tetraploids had lower NPQ values than diploids, indicating higher ability to minimize heat damage and efficiently utilize absorbed light energy.

After thorough phenotypic and physiological evaluation, the tetraploids obtained exhibited obvious advantages in some traits. One the one hand, these plants can be directly applied. One the other hand, they can be used for further breeding, for example, to cross with diploids or tetraploids in efforts to obtain triploids or tetraploids with traits that provide higher economic value than the parents.

## Materials and methods

### Plant material

We cultivated *A. roxburghii* plants, vegetatively propagated *in vitro*, in the Chinese Medicinal Plants Garden on the South China Agricultural University campus. Tetraploid plants were obtained by colchicine treatment of diploid nodal explants, following the process described by Wang et al. (31). The ploidy level was estimated using a flow cytometer (CyFlow^®^ Ploidy Analyzer, Sysmex, Japan) with a CyStain UV OxProtect kit (Jiyuan Biotech, China) according to Cai and Kang (32) and diploid *A. roxburghii* plants were used as controls.

### Phenotypic characterization

We measured the height, stem diameter, internode length, leaf area and thickness, stomata, root system, and whole weight of sampled plants. We also determined their fresh weight using an electronic balance (Sartorius, China), and their dry weight after drying to a constant weight at 65^°^C. Leaf thickness was analyzed using a digital caliper (Meinaite, China). We measure the size of mature leaves with a CL-203 laser area meter (CID, USA). The root system, plant height, and stem diameter were scanned using a WinRhizo Pro LA2400 system (Regent, Canada). Stomatal areas were measured in fresh fully developed and healthy adult leaf portions and observed under a microscope (Nikon Eclipse Ni-U, Japan).

### Chlorophyll fluorescence analysis

Chlorophyll fluorescence parameters of the second and third leaves from the top of selected plants were measured with a dual PAM-100 fluorometer (Heinz-Walz, German), using red actinic light and a red measuring beam for fluorescence. After 30 min dark adaptation, minimal fluorescence (F_0_), the maximum fluorescence (Fm), and maximal level of P700 signal (P_*m*_) were determined according to standard Dual-PAM-100 protocols. We calculated the maximum efficiency of PSII photochemistry (Fv/Fm), quantum yield of PSI Y(I), effective photochemical quantum yield of PSII Y(II), electron transport rates of PSI and PSII (ETR I and II, respectively), non-photochemical quantum yields of PSI due to acceptor-side limitation Y(NA) and donor-side limitation Y(ND), and non-photochemical quenching (NPQ) following Hikosaka (33).

### Photosynthetic pigment content

Second leaves from the top of sampled plants were collected for determination of photosynthetic pigments. The fresh leaves were ground with 80% (V_*acetone*_/V_*water*_) acetone at room temperature then centrifuged (5,000 g, 5 min). The supernatant’s absorbance was measured with a Varioskan LUX spectrometer (Thermo Scientific, Finland) at 663.2 and 646.8 nm. Chl a and Chl b concentrations were then calculated using equations of Porra (34).

### Free amino acids and mineral elements analysis

Free amino acids were determined following Liu et al. (35) using a L-8900 automatic amino acid analyzer (Hitachi, China). The concentrations of macro nutrients (K, Ca, Mg, Fe, Mn, Zn, and Cu) were quantified by a 220FS flame atomic absorption spectrometer (Varian, USA), following Li et al. (36). Micro nutrients (Cr, Co, and Ni) were determined using a Zeenit 650P graphite furnace atomic absorption spectrometer (Analytik Jena, Germany) according to the method described by Manjusha et al. (37).

### Determination of main bioactive secondary metabolite production

Kinsenoside, narcissin, rutin, quercetin, and isorhamnetin were extracted and analyzed. For this, samples were oven-dried and then ground to 100-mesh size. A sample of 10 mg powder was weighed and soaked in 1.2 mL of 70% (V_*methanol*_/V_*water*_) methanol. After ultrasonic extraction for 30 min, each resulting suspension was centrifuged (10,000 g, 10 min), then filtered with a 0.22 μm syringe filter, placed in a sample vial and stored at 4^°^C before measurement. The samples were analyzed using a HPLC-ESI-MS/MS system (Agilent 1290–6470, USA) equipped with a 2.1 × 50 mm, 1.8 μm C18 reverse phase column. The mobile phase consisted of water with 0.2% formic acid (solvent A) and acetonitrile (solvent B) at a flow rate of 0.4 mL/min, starting with a 1 min hold at 90% A followed by a linear 3-min gradient from 90 to 10% A, then a 1 min hold at 10% A, a 1 min linear rise back to 90% A and 4 min hold at 90% A for re-equilibration before the next injection. The column temperature was 40^°^C and injection volume 2 μL. The MS system was a 6470 Triple Quadrupole mass spectrometer, with the following settings: dry temperature 300^°^C, gas flow rate 6 L/min; nebulizer pressure 45 psi, sheath temperature 350^°^C, sheath gas flow rate 10 L/min, and capillary voltage 4 kV. Contents of analytes in samples were determined using the external standard method, with peak height as the quantitative parameter.

### Statistical analysis

Three typical individuals of each ploidy and three biological replicates, were used in all the experiments. Data acquired in the experiments were sorted using Microsoft Office Excel (Microsoft Corp., Redmond, WA, USA), then statistical analyses were performed with SPSS software (Version 26.0; SPSS Inc., Chicago, IL, USA). Student’s *t*-tests were applied to assess the significance of differences between parameters of diploid and tetraploid plants. *P*-values ≤ 0.05 were considered statistically significant.

## Data availability statement

The raw data supporting the conclusions of this article will be made available by the authors, without undue reservation.

## Author contributions

YY and JZ conceived and designed research. XH, KO, YL, and GX conducted the experiments, collected, and analyzed the data. XH and JZ wrote the manuscript. YY obtained the fundings. All authors contributed to the article and approved the submitted version.
